# Distinct characteristics of the DNA damage response in mammalian oocytes

**DOI:** 10.1038/s12276-024-01178-2

**Published:** 2024-02-14

**Authors:** Jiyeon Leem, Crystal Lee, Da Yi Choi, Jeong Su Oh

**Affiliations:** Department of Integrative Biotechnology, Sungkyunkwan University, Suwon, South Korea

**Keywords:** Checkpoint signalling, Meiosis, Oogenesis

## Abstract

DNA damage is a critical threat that poses significant challenges to all cells. To address this issue, cells have evolved a sophisticated molecular and cellular process known as the DNA damage response (DDR). Among the various cell types, mammalian oocytes, which remain dormant in the ovary for extended periods, are particularly susceptible to DNA damage. The occurrence of DNA damage in oocytes can result in genetic abnormalities, potentially leading to infertility, birth defects, and even abortion. Therefore, understanding how oocytes detect and repair DNA damage is of paramount importance in maintaining oocyte quality and preserving fertility. Although the fundamental concept of the DDR is conserved across various cell types, an emerging body of evidence reveals striking distinctions in the DDR between mammalian oocytes and somatic cells. In this review, we highlight the distinctive characteristics of the DDR in oocytes and discuss the clinical implications of DNA damage in oocytes.

## DNA Damage in Oocytes: A Key Player in Infertility and Birth Defects

Understanding how our cells fix DNA damage is key to understanding why certain diseases, like cancer, happen. Researchers conducted a review, to create a clearer picture of DNA repair in oocytes. They discovered that oocytes have a unique method of handling DNA damage compared to other cell types. The study also noted that as women age, their oocytes become less skilled at repairing DNA, which can affect fertility. The researchers emphasized that oocytes have a strong system for fixing DNA damage, which is crucial for preventing chromosomal abnormalities in embryos. They concluded that understanding these repair mechanisms better could lead to advancements in fertility treatments and preserving reproductive health. Future research could further unravel these processes, potentially leading to new ways to help individuals facing fertility challenges.

This summary was initially drafted using artificial intelligence, then revised and fact-checked by the author.

## Introduction

DNA, the fundamental molecule of heredity, is considered a remarkably stable entity. However, its inherent stability does not render it impervious to the potential threats posed by numerous endogenous and exogenous assaults during cellular processes. Therefore, cells have evolved a specialized defense mechanism to safeguard genomic integrity, which is collectively termed the DNA damage response (DDR)^1,2^. DDR signaling coordinates an intricate network of various cellular processes, such as cell cycle arrest, DNA repair, apoptosis, and senescence. Defects in the DDR may lead to genomic instability, contributing to tumorigenesis and several disorders^3–5^. The DDR encompasses multiple pathways, each involving the detection of and response to specific types of DNA lesions, such as intrastrand crosslinks, base modifications, single-strand breaks (SSBs), and double-strand breaks (DSBs). Given the heightened risk of DSBs among diverse DNA lesions, we will focus on the DDR associated with DSBs in this review.

In eukaryotic cells, cellular responses to DSBs are coordinated through highly conserved signaling cascades controlled by the kinase ataxia telangiectasia mutated (ATM)^6–8^. ATM is activated at DSB sites through its interaction with the MRE11-RAD50-NBS1 (MRN) complex, initiating a series of phosphorylation events^7^. Once activated, ATM phosphorylates several substrates, including the histone variant H2AX (referred to as γ-H2AX), the MRN complex, and the downstream checkpoint kinase 2 (CHK2)^8,9^. Activated CHK2, in turn, phosphorylates multiple proteins involved in cell cycle progression or apoptosis, such as the p53 tumor suppressor protein and cell division cycle 25 (CDC25) phosphatase^10,11^. Recent studies have highlighted an additional layer of complexity by revealing that CHK1 is also activated by ATM^12,13^. Thus, ATM-CHK1/2 cascade events stimulate the activity of WEE1 and maintain a high level of inhibitory phosphorylation of cyclin-dependent kinase 1 (CDK1), preventing cell cycle progression in the presence of unrepaired DNA damage^8,11,14^. Furthermore, several proteins, including mediator of DNA damage checkpoint 1 (MDC1), p53-binding protein 1 (53BP1), breast cancer susceptibility gene 1 (BRCA1), and the ubiquitin ligases ring finger containing nuclear proteins 8 (RNF8), are recruited to DSB sites. These proteins play a crucial role in coordinating DSB repair by further recruiting downstream repair factors^3,5,14^.

Women are born with a finite pool of primordial follicles, which serve as the source of oocytes throughout their reproductive life^15^. During this prolonged period, mammalian oocytes within the ovary remain arrested at the beginning of the first meiosis, rendering them particularly susceptible to various endogenous and exogenous insults that may cause DNA damage^16^. The accumulation of DNA damage can result in serious chromosomal abnormalities in embryos, ultimately leading to abortion or infertility^17,18^. Therefore, understanding the DDR in oocytes is of paramount importance in reproductive biology and fertility preservation. While extensive research has shed light on DDR mechanisms in somatic cells, such as in the context of cancer, we are only now beginning to understand the complex array of the DDR in mammalian oocytes. In this review, we highlight the distinct characteristics of the DDR in mammalian oocytes and discuss the clinical implications of DNA damage in oocytes, particularly in relation to decreased fertility.

## Oocyte development and maturation

Mammalian oogenesis begins during early embryogenesis with the emergence of primordial germ cells^19,20^. These cells migrate to the genital ridge and develop into primary oocytes surrounded by somatic cells, thus forming primordial follicles^20,21^. During this process, primary oocytes enter meiosis at the prophase stage, a crucial phase for genetic dynamics in which homologous chromosomes pair and recombine^19^. After birth, oocytes reach the diplotene stage of prophase and remain arrested in the ovaries for several months to decades before resuming their meiotic cycle^22^. These oocytes are characterized by a large nucleus known as the germinal vesicle (GV). Following the hormonal surge at puberty, oocytes resume meiosis by undergoing GV breakdown (GVBD), leading to chromatin condensation. After GVBD, oocytes enter the metaphase I (MI) stage, where chromosomes randomly align to the cell equator and kinetochores are captured by dynamically expanding and contracting microtubules, ensuring correct biorientation^23^. This strategic division ensures that sister kinetochores form individual structures within a pair that can form independent attachments to spindle kinetochore fibers from the same pole of the spindle, while homologous kinetochores attach to the opposite pole, reducing the ploidy of cells in meiosis I^24–26^. In late MI, the meiotic spindle migrates to the nearest cortex via a mechanism that is regulated by actin filaments^27^. Simultaneously, the kinetochore-microtubule (K-MT) attachments are fully completed, leading to the separation of homologous chromosomes. In contrast to mitosis, which yields two daughter cells of equal sizes, asymmetric meiosis results in one daughter cell acquiring the majority of the cytoplasm, while the remaining cell, called a polar body, receives a minimal amount of cytoplasm^28^. After extruding the first polar body, oocytes immediately enter the second meiosis and remain arrested at the metaphase II (MII) stage while awaiting fertilization by sperm. Oocytes are then ready for ovulation in the MII stage, where they are released into the oviduct through a series of dynamic tissue remodeling events. Following fertilization, arrested meiosis is reinitiated to extrude the secondary polar body, leaving behind a haploid female pronucleus^29^.

## Distinct features of the DNA damage response in oocytes

Although the DDR is a highly conserved cellular process, mounting evidence points to significant differences between the DDR in mammalian oocytes and that in somatic cells. In this section, we highlight the distinct features of the DDR in mammalian oocytes.

### Decreases in apoptosis during follicular development

**Fig. 1:**
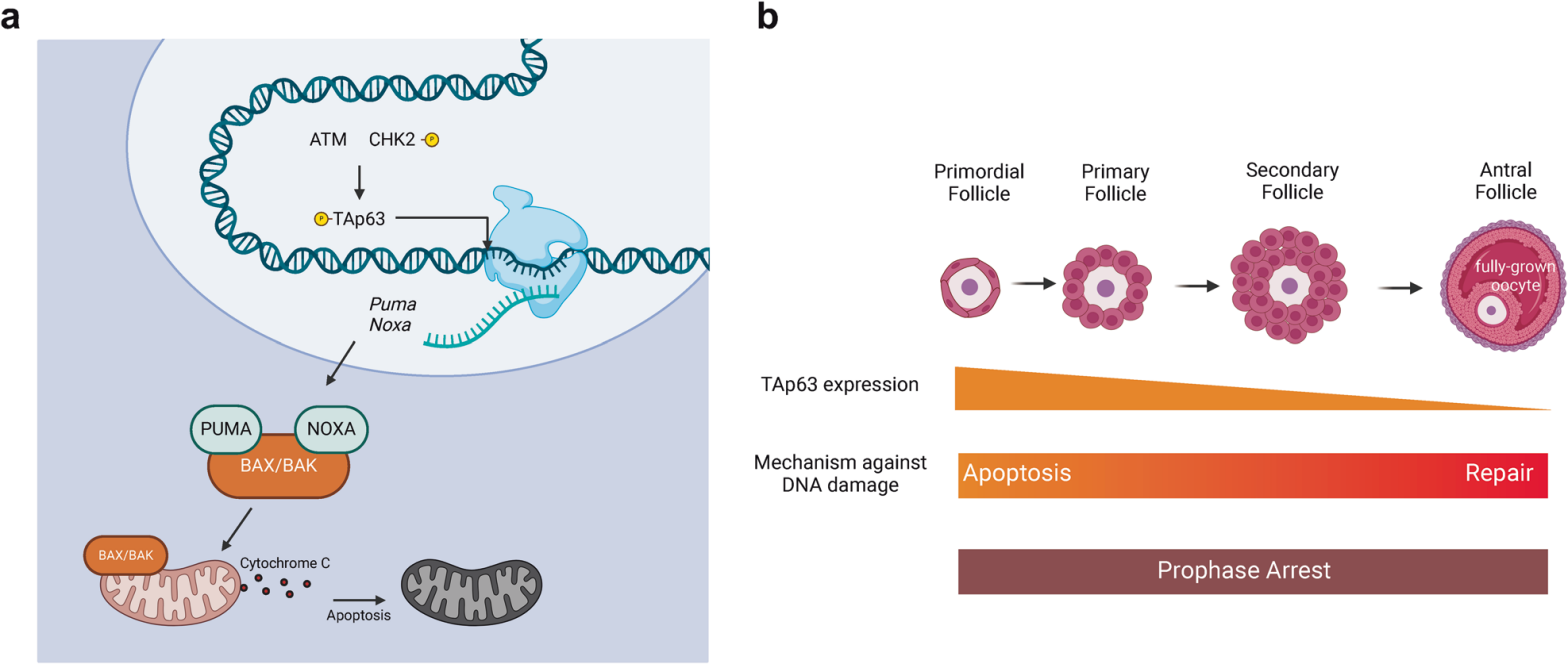
DNA damage-induced apoptosis in oocytes. **a** TAp63-induced apoptosis in oocytes from primordial and primary follicles. DNA damage triggers the activation of TAp63, which in turn mediates oocyte apoptosis by inducing the transcription of PUMA and NOX and the subsequent inhibition of BAX and BAK. **b** Decreased apoptosis during follicular development. During the early stages of follicular development, both primordial and primary follicles highly express TAp63, which allows them to respond to DNA damage through apoptosis. However, as follicles progress to the late antral stage, TAp63 expression diminishes. Consequently, fully grown oocytes become less prone to undergo apoptosis after DNA damage.

Oocytes within primordial follicles readily undergo apoptosis by triggering the transactivating p63 (TAp63)-dependent signaling pathway in response to DNA damage^30–33^. As a homolog of p53, TAp63 is highly expressed and orchestrates apoptosis in the oocytes of primordial follicles^34,35^. When DNA damage occurs, TAp63 is activated through the sequential phosphorylation mediated by the canonical ATM-CHK2 signaling pathway^36^. Activated TAp63, in turn, induces the transcription of the BH3-only proapoptotic BCL2 family proteins PUMA and NOXA^30^. Like all BH3-only proteins, both proteins initiate apoptosis by activating the proapoptotic BCL2 family proteins BAX and BAK^16,37^. The subsequent translocation of BAX/BAK to mitochondria triggers apoptotic reactions, such as cytochrome c release and caspase 9 activation^38^. This cascade of events ultimately leads to oocyte apoptosis (Fig. 1a). Indeed, the oocytes of TAp63-null mice are resistant to chemotherapeutic agents, including irradiation and cisplatin, at doses that can kill the oocytes of wild-type and p53-null mice, indicating that TAp63 regulates oocyte apoptosis^31,39^. Similarly, PUMA- or PUMA/NOXA-null mice also exhibit a high capacity for survival against genotoxins, which reduces the amount of cell residue in wild-type oocytes^30,31^. Thus, TAp63 and PUMA/NOXA play crucial roles in initiating apoptosis during the primary phase of oocyte development.

While oocytes derived from PUMA or PUMA/NOXA knockout models exhibit high resistance to apoptosis, they maintain fertility and produce healthy offspring^30^. These findings suggest that these cells have a highly efficient DNA repair mechanism and normal oocyte functionality. Notably, TAp63 expression is high in primordial/primary follicles and gradually decreases as follicles develop^31,40^. This decrease in TAp63 expression reduces the capacity of oocytes to respond to apoptotic signals. Consequently, fully grown oocytes in late-stage follicles can survive and continue their development, even in the presence of accumulated DNA damage^31^. During this period, the DNA repair pathway of oocytes becomes the main safeguard against DNA damage, effectively preventing DNA accumulation (Fig. 1b). Considering that female germ cells are finite from birth, the reason why apoptosis is favored at the primary stage but then this preference gradually switches to the DNA repair pathway is unclear. Since the reproductive lifespan of a female is established during fetal development and persists until menopause without generating new pools^15^, it is crucial to preserve high-quality oocytes from the initial stages. In this context, apoptosis during the early development of follicles and oocytes may serve as the primary checkpoint for identifying and eliminating severely damaged cells instead of attempting to repair them. This process also acts as a proactive measure to reduce the potential transmission of genetic mutations to future generations. However, once oocytes have fully grown and are ready to resume meiosis, they become a valuable resource for future fertilization. Thus, by prioritizing DNA repair over apoptosis in these mature oocytes, the female reproductive system may maximize the chances of producing offspring while maintaining genomic integrity. Understanding the mechanism underlying the reduction in apoptosis during follicle/oocyte development may provide valuable insights into preserving high-quality oocytes throughout the reproductive lifespan of females and ensuring that healthy genetic material is provided for embryos; however, a detailed investigation is still needed.

### Lack of a robust G2/M DNA damage checkpoint

**Fig. 2:**
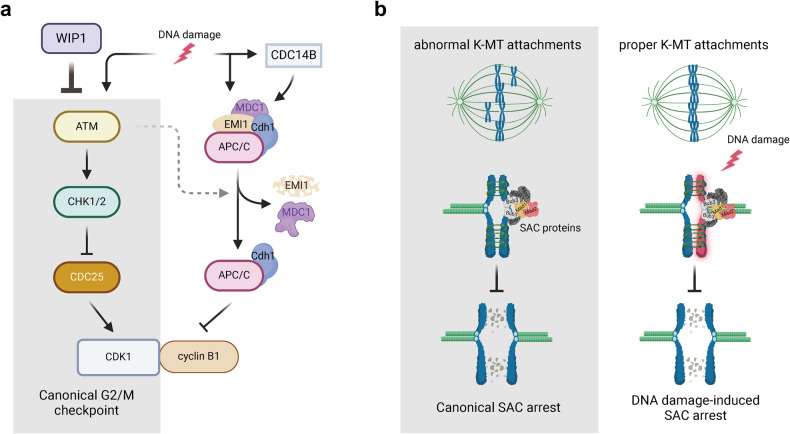
DNA damage checkpoints in oocytes. **a** Lack of a robust G2/M DNA damage checkpoint. The canonical ATM-CHK1/2-CDC25 pathway is downregulated in fully grown oocytes and is partly influenced by WIP1 activity. Instead, oocytes use a noncanonical G2/M DNA damage checkpoint, which is associated with the regulation of APC/C-Cdh1 activity. DNA damage induces EMI1 degradation and MDC1 dissociation from APC/C-Cdh1, as well as CDC14B activation, which eventually leads to an increase in APC/C-Cdh1 activity. This, in turn, promotes cyclin B1 degradation, subsequently delaying GVBD. **b** DNA damage-induced SAC arrest. In general, the SAC is activated in the presence of unattached kinetochores, which leads to the inhibition of APC/C and subsequent anaphase onset. However, in oocytes with DNA damage, the SAC is activated independently of unattached kinetochores during meiosis I.

The cell cycle is a highly regulated process that ensures accurate duplication and segregation of genetic material^41–43^. The G2/M DNA damage checkpoint plays a critical role in this process by monitoring DNA integrity before cells enter mitosis, acting as a protective barrier against mitotic entry in cells with DNA damage^44,45^. By allowing sufficient time for DNA repair and genomic stability maintenance, this checkpoint helps to safeguard genomic integrity. Because the stage of prophase arrest of oocytes corresponds to the G2/M transition of somatic cells^46^, the mechanisms governing the G2/M checkpoint in response to DNA damage in oocytes were initially assumed to be similar to those in somatic cells. However, recent studies have reported that DNA damage, which typically causes G2 arrest in somatic cells, does not arrest fully grown mouse oocytes in prophase unless the damage is severe, suggesting that oocytes lack a robust G2/M checkpoint^16,47–50^ (Fig. 2a). This inability to establish G2/prophase arrest is due to the lack of ATM kinase activation, which indicates low ATM expression. Only high levels of DNA damage can activate ATM in mouse oocytes, leading to CHK1-dependent inhibitory phosphorylation of CDC25 and prevention of meiosis resumption^50^. Prophase arrest has also been observed in other mammalian species, as human and porcine oocytes do not appear to initiate a checkpoint following exposure to high levels of genotoxic agents such as etoposide^51,52^.

However, why oocytes exhibit a deficiency in the G2/M DNA damage checkpoint remains unclear. However, the inhibition of wild-type p53-induced phosphatase 1 (WIP1) following DNA damage increases the level of γ-H2AX through ATM activation, effectively establishing the G2/M checkpoint in response to DNA damage. This, in turn, leads to the delay or inhibition of meiotic resumption^53^. These findings suggest that oocytes can mount a robust G2/M DNA damage checkpoint, but this response is suppressed by WIP1 activity. Given that WIP1 is generally downregulated during mitosis^54^, why oocytes express WIP1 and suppress ATM activity during prophase arrest is unclear. One speculation is that ATM suppression may be beneficial for facilitating programmed DSBs for homologous recombination (HR) during early prophase in oocytes. In addition, there is evidence of a noncanonical G2/M DNA damage checkpoint in mouse oocytes^55^. This alternative pathway is activated through increased anaphase-promoting complex/cyclosome (APC/C) activity, which in turn promotes the proteolysis of cyclin B1. This effect is attributed to enhanced protein phosphatase cell division cycle 14B (CDC14B) activity and reduced activity of the APC/C inhibitor early mitotic inhibitor 1 (EMI1)^55^. However, it should be noted that the APC/C-mediated G2/M checkpoint does not occur rapidly in response to DNA damage. In line with this, it has been reported that MDC1 plays a crucial role in regulating the APC/C-Cdh1-mediated G2/M checkpoint in mouse oocytes^56^. MDC1 directly interacts with APC/C-Cdh1 to exert this control. Notably, this novel interaction between MDC1 and APC/C-Cdh1 is disrupted by DNA damage and increases APC/C-Cdh1 activity, which in turn delays meiotic resumption^56^. In addition to the above mechanisms, the G2/prophase DNA damage checkpoint in oocytes becomes more robust when the oocyte is surrounded by a layer of cumulus cells. Studies have indicated that DNA damage promotes cAMP production in cumulus cells, which can be transferred to oocytes through the gap junctions connecting oocytes and cumulus cells^57^. This communication may contribute to the strengthening of the G2/M checkpoint in oocytes. Collectively, these findings indicate that mammalian oocytes do not immediately respond to DNA damage via a robust checkpoint but instead mount a weak and less efficient G2/M DNA damage checkpoint, which involves APC/C-Cdh1-mediated proteolysis.

### DNA damage-induced spindle assembly checkpoint (SAC)

The lack of a robust G2/M checkpoint indicates that fully grown oocytes with DNA damage can progress beyond GVBD and undergo meiotic maturation if the damage is not severe. However, oocytes exposed to DNA-damaging reagents do not reach the MII stage and instead remain arrested at the MI stage^49,58,59^. Interestingly, this MI arrest is not dependent on the canonical ATM/ATR pathways but is caused by the activation of the spindle assembly checkpoint (SAC)^49,59^.

SAC activity coordinates chromosome segregation by ensuring proper K-MT attachment^60,61^. When abnormal K-MT attachments are detected, the SAC is activated, resulting in a cascade of events that ultimately lead to the inhibition of anaphase onset. Unattached kinetochores bind the SAC proteins mitotic arrest deficiency 1 and 2 (MAD1 and MAD2) to form a platform allowing further recruitment of MAD2. Activated MAD2 is then released into the cytoplasm, creating a reversible ‘wait-anaphase’ signal by isolating CDC20, resulting in the inhibition of the APC/C^62–64^. This, in turn, stabilizes substrates of the APC/C, including Aurora kinases, Polo-like kinase-1 (PLK1), cyclin B1, and Securin, thereby maintaining CDK1 activity, inhibiting Separase, and preventing the onset of anaphase^63,65,66^. However, DNA damage-induced SAC activity seems to differ from canonical SAC activity, which is typically triggered by unattached kinetochores (Fig. 2b). Indeed, there is no obvious loss of K-MT attachments or tension across bivalents in oocytes exposed to etoposide^64^. Moreover, if the level of DNA damage is considerably low, oocytes can still extrude from the polar body, albeit with a delay in the first polar body extrusion, possibly resulting in aneuploidy^60^. Furthermore, recent studies have reported that exogenous DNA damage induction does not activate the SAC in human oocytes; thus, this damage occurs throughout meiotic maturation, causing chromosome damage and aberrant spindles in mature MII oocytes^52^. Indeed, it was reported that anaphase onset was dependent on the loss of CDK1 activity rather than nonaligned bivalents^63^. Thus, it is likely that a few abnormal K-MT attachments did not induce robust SAC activation in mammalian oocytes. A threshold number of abnormal K-MT attachments may be required before the SAC can be successfully activated, and cohesin is crucial for SAC activity in MI oocytes in that cohesin deterioration compromises the SAC^67^.

Compromised SAC efficiency has been reported in aging oocytes^68^. Therefore, DNA damage may impose severe chromosomal anomalies in females of advanced reproductive age, which is detrimental to embryonic development. However, no significant recruitment of SAC components occurs on MII kinetochores after DNA damage^64^. Thus, DNA damage does not induce SAC activation at the MII stage, although oocytes can normally activate the SAC in response to MII spindle disruption or chromosome misalignment. As mentioned, oocytes are especially prone to DNA damage accumulation over time. Thus, it is important that oocytes detect and delay meiotic maturation to provide sufficient time for DNA damage to be repaired. In this regard, the SAC is of particular importance because it is the final major checkpoint ensuring oocyte quality and genome integrity before oocytes are ready for fertilization.

## DNA repair during meiotic maturation

Despite being integral to cellular survival, DNA repair occurs primarily in interphase and is repressed during mitosis^69–71^. Nevertheless, emerging evidence suggests that oocytes have the capacity to repair DSBs during meiosis. Here, we discuss the mechanisms of DNA repair, particularly those related to DSBs, in oocytes during meiotic maturation.

### Recruitment of BRCA1 and 53BP1 at DSB sites during meiosis

In response to DNA damage, the initial events, including H2AX phosphorylation and MDC1 recruitment, seem to be intact, but downstream repair factors, such as RNF8, BRCA1, and 53BP1, are not successfully recruited to DSB sites during mitosis^69,72,73^. This failure is attributed to mitosis-specific phosphorylation of RNF8 and 53BP1. Specifically, 53BP1 is phosphorylated at T1609 and T1618 by CDK1 and PLK1, which hinders its binding to γ-H2AX^74,75^. Furthermore, RNF8 is phosphorylated at T198 by CDK1, which prevents its interaction with MDC1 and thereby impairs its recruitment to DSB sites^76^. Instead, MDC1 recruits TOPBP1 to mitotic DSB sites and tethers broken chromosome ends by forming a filamentous structure that links MDC1 foci^73^. This intricate mechanism allows mitotic cells to efficiently mark DSB sites for repair during the subsequent G1 phase. In stark contrast, recent reports have demonstrated that oocytes can recruit BRCA1 and 53BP1 and repair DSBs via both the HR and NHEJ repair pathways during meiosis^77–79^. Although the mechanisms facilitating the recruitment of BRCA1 and 53BP1 to DSBs in oocytes are not fully understood, it can be speculated that mitosis-specific phosphorylation of RNF8 and 53BP1 could be temporarily and/or spatially dampened after DNA damage in oocytes during meiosis. Indeed, PLK1 was found to be inactivated at the spindle pole in oocytes after the induction of DSBs^77^.

### Distinct roles of HR and NHEJ during meiotic maturation

**Fig. 3:**
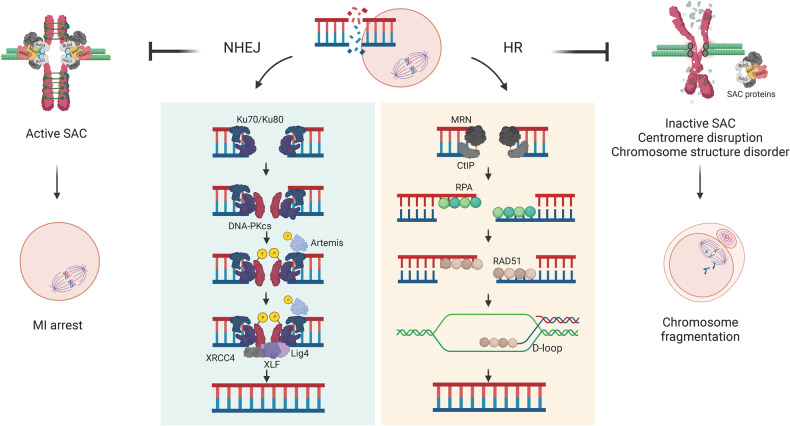
Distinct roles of NHEJ and HR during meiotic maturation. Both the HR and NHEJ repair pathways function separately yet together during meiotic maturation. The details of each pathway are illustrated and highlighted in the boxed areas. When NHEJ is inhibited during meiotic maturation, oocytes with DNA damage exhibit increased DNA damage levels and arrest at the MI stage via SAC activation. On the other hand, HR suppression decreases the integrity of the centromere, causing centromere disruption and SAC inactivation. This further damages chromosome structure and causes chromosome fragmentation during chromosome segregation.

DSBs are repaired primarily through two major mechanisms: nonhomologous end joining (NHEJ) and homologous recombination (HR)^80–83^. NHEJ begins with the recognition of DSB ends by a heterodimer consisting of Ku70 and Ku80, which subsequently recruits the DNA-dependent protein kinase catalytic subunit (DNA-PKcs)^84,85^. DNA-PK subsequently activates and phosphorylates itself as well as other end-processing enzymes, such as Artemis^84,85^. The DNA ligase IV complex, including X-ray repair cross complementing 4 (XRCC4) and XLF, then performs the critical ligation step for either strand of DSB^84,85^. In contrast, the HR pathway is initiated when the CtBP-interacting protein (CtIP) interacts with the MRN complex, forming short single-stranded DNA (ssDNA) at the DSB ends^86,87^. After generating an ssDNA overhang, the replication protein A (RPA) complex and downstream components are recruited for 3′-ssDNA extension, and the termination of end resection is facilitated by RAD51-RPA switching^88^. The resected 3′-RAD51-ssDNA presynaptic filament then invades homologous double-stranded DNA (dsDNA), generating a displaced strand (D-loop) intermediate^86,87^. The displacement of RAD51 exposes the 3′ end of the intermediate for DNA extension by DNA polymerase, and the invading strand is displaced and resolved with complementary templates^87^ (Fig. 3).

As NHEJ does not require a homologous template for repair, it is generally considered error prone and dominant throughout interphase^88^. However, it was also reported that “clean” DSBs with complementary overhangs can be accurately repaired by the classic NHEJ pathway, showing nearly equal efficiencies in the presence of a homologous template^84,89^. In oocytes, NHEJ was found to be the preferential repair pathway during MII, as the inhibition of NHEJ-related elements was associated with increased DNA damage signals and decreased developmental competence^78,79^. Moreover, oocytes exposed to etoposide at the prophase stage undergo an increase in SAC-mediated MI arrest upon NHEJ inhibition, along with partially impaired K-MT attachment^78^ (Fig. 3). This finding was in contrast with reports showing that damage-induced SAC arrest is independent of aberrant K-MT attachments^64^, suggesting that NHEJ plays a role in maintaining the DNA damage repair capacity of oocytes, subsequently determining meiotic maturation completion. On the other hand, the HR pathway requires a homologous strand as a template to efficiently repair DSBs and is generally considered to be error free and preferable in the S and G2 phases^84,88^. Considering the difference in the mechanisms involved between the HR and NHEJ pathways, it can be speculated that each pathway plays a differential role in the DDR throughout cell cycle progression. Indeed, there is evidence showing that each repair pathway plays distinct roles in repairing DSBs during meiotic maturation^78^. In contrast to NHEJ, when HR is inhibited, the oocyte cell cycle is not affected, despite significant chromosome fragmentation possibly resulting from compromised centromere integrity, suggesting crosstalk between the highly repetitive sequences in the centromere and the HR machinery^78^ (Fig. 3). HR has been shown to play a role in centromere protection in somatic cells when DSBs at centromeres in G1 are repaired by the HR machinery; thus, HR inhibition results in centromeric instability and chromosomal translocation^90^.

The DSB repair pathway appears to switch from HR to NHEJ as oocytes mature from GV to MII. Although the crosstalk mechanism between HR and NHEJ and their transition are unclear, the accessibility of the components of each pathway at different cell stages may determine the preferred DNA damage repair mechanism during oocyte maturation^88,91^. The initiation of end resection generally guides the cell to the HR pathway, as Ku has limited binding affinity for long ssDNA overhangs^92^. HR was also reported to be more efficient in the absence of NHEJ components, suggesting a compensatory role for HR when the other repair pathway is unavailable^93^. In addition to these widely studied pathways, a third repair pathway, microhomology-mediated end joining (MMEJ), has received increasing interest in DNA damage repair. In MMEJ, short homologous sequences (microhomologies) generated by DNA end resection are annealed to align DSB ends before ligation, thus possibly resulting in deletions flanking the break^91,94^. However, further research is needed to fully understand the roles and interactions between these DSB repair pathways in safeguarding the genome integrity of oocytes.

### Role of spindle microtubules in DNA repair during oocyte meiosis

Chromatin undergoes constant movement and plastic reorganization within the nucleus^95^. Intriguingly, this dynamic movement of chromatin becomes more pronounced in response to DNA damage^96^. Indeed, dynamic reorganization of chromatin has been observed at DSBs^97,98^. This enhanced mobility is thought to facilitate the search for donor sequences needed by HR or to enable efficient access to repair factors, thereby promoting effective DNA repair^96–98^. The cytoskeleton plays a critical role in driving chromatin movement at damaged sites. The coordination of chromatin and the cytoskeleton contributes to the proper positioning of damaged DNA and the efficient repair of damaged DNA^99,100^. Recent studies in yeast and mammalian cells have demonstrated that cytoplasmic actin and microtubules trigger widespread changes in chromatin structure in response to DNA damage^27,101,102^. Furthermore, γ-tubulin has been shown to interact with RAD51 and form a nuclear complex with BRCA1 following DNA damage, implying that there is a mutual interaction between DNA repair and microtubule networks^103^. Therefore, interplay between chromosomes and the cytoskeleton is implicated in maintaining genomic stability against DNA damage. However, this interplay appears to be restricted to interphase, and whether it is effective during mitosis has not been determined.

**Fig. 4:**
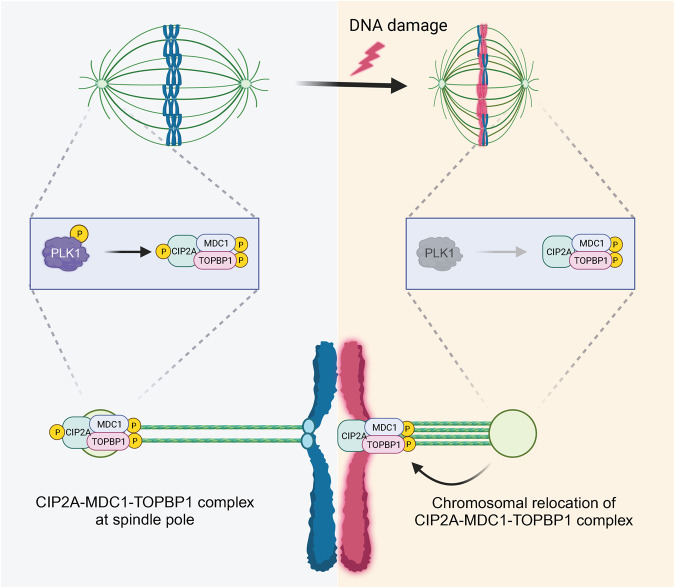
DSB repair during meiosis I. In response to DNA damage, a series of coordinated events take place in MI oocytes to facilitate DSB repair. DNA damage triggers rapid shrinkage and stabilization of spindle microtubules and concomitant and transient inactivation of PLK1 at spindle poles. This leads to the dephosphorylation of CIP2A in complex with MDC1 and TOPBP1 and the subsequent recruitment of these proteins to chromosomes from the spindle poles. This mechanism ensures the efficient and accurate repair of DNA damage, preserving genomic integrity during oocyte meiosis.

A recent study revealed novel dynamics in spindle microtubules in response to DNA damage during meiosis in mouse oocytes^77^. After DNA damage, the spindle microtubules rapidly shrink and are stabilized. This process involves the inactivation of PLK1 at the spindle poles, leading to the dephosphorylation of the cancerous inhibitor of protein phosphatase 2 A (CIP2A) at S904. Dephosphorylated CIP2A, along with p-MDC1 and p-TOPBP1, moves along spindle microtubules and is recruited to chromosomes through kinetochores and centromeres. This chromosomal relocation ensures the recruitment of downstream repair factors such as BRCA1 and 53BP1 for DNA repair during oocyte meiosis (Fig. 4). Although the biological significance of spindle shrinkage and stabilization has not been determined, many proteins involved in DDR pathways, including the CIP2A-MDC1-TOPBP1 complex, are associated with spindle microtubules or are localized at spindle poles^56,77,104^. This association might facilitate DNA repair by bringing DDR factors closer to chromosomes during oocyte meiosis. Surprisingly, a study revealed that neither ATM, PLK1, nor K-MT attachment was the driving force behind spindle changes in response to DNA damage in oocytes^77^. Given the intimate interaction between actin filaments and microtubules during spindle assembly in oocytes^105^, it is reasonable to postulate that actin filaments are involved in the process governing DSB-induced spindle changes. Indeed, concomitant actin filament assembly has been reported after DNA damage in somatic cells^106^. However, further studies on the mechanism of chromosome cross-talk with spindle poles in response to DNA damage in oocytes are needed.

## DNA damage and oocyte quality

DNA damage in oocytes can result in meiotic errors, such as chromosomal nondisjunction or improper recombination, leading to aneuploidy, which is characterized by an abnormal number of chromosomes in the resulting embryo^107^. Aneuploid embryos have reduced implantation potential and are associated with a higher risk of miscarriage or the birth of a child with genetic abnormalities, including Down syndrome^108–110^. Moreover, DNA damage induces epigenetic changes in oocytes, and these changes affect the regulation of gene expression in the resulting embryos. Aberrant epigenetic patterns resulting from DNA damage in oocytes can lead to developmental abnormalities and increased susceptibility to diseases later in life^109,111–113^. Furthermore, cancer therapies, such as chemotherapy and radiation, can induce DNA damage not only in cancer cells but also in healthy cells, including oocytes. The impact of cancer treatments on oocyte quality and fertility depends on factors such as the type, dose, and duration of therapy. The DNA damage incurred by oocytes during cancer treatment can result in temporary or permanent loss of fertility, premature ovarian insufficiency, and an increased risk of genetic abnormalities in offspring conceived posttreatment^33,114,115^.

Mitochondria play an essential role in providing the energy and building blocks necessary for transcription and translation during oocyte maturation and fertilization and early embryo development. Moreover, mitochondria coordinate numerous cellular processes, including apoptosis and reactive oxygen species (ROS) homeostasis^116,117^. Mitochondrial dysfunction has been found to be closely associated with compromised oocyte quality, reduced embryo viability, and an increased risk of infertility^112,113^. Given that mitochondria contain their own DNA, it is notable that DNA damage impairs mitochondrial function and thereby decreases oocyte quality. Notably, both the number and activity of mitochondria decrease with advancing age^113^. Thus, mitochondrial supplementation and replacement, aimed at enhancing, reversing, or slowing oocyte aging, have recently emerged as therapeutic strategies^118–125^.

Oxidative stress is one of the main causes of DNA damage and contributes to oocyte aging and decreased oocyte quality^126–128^. This challenge becomes more pronounced during assisted reproductive technology (ART) procedures, such as in vitro fertilization and intracytoplasmic sperm injection, where reactive oxygen species (ROS) production is unavoidable during in vitro culture^129–131^. Therefore, numerous studies have focused on protecting oocytes from ROS to preserve oocyte quality and enhance fertility. The most common approach is the use of antioxidants to mitigate DNA damage. Antioxidants, such as glutathione (GSH), melatonin, vitamin E, resveratrol, and coenzyme Q10 (CoQ10), have been shown to have protective effects against DNA damage in oocytes by scavenging ROS and maintaining redox balance^132–138^. The incorporation of antioxidant supplementation strategies holds great potential for improving oocyte quality, enhancing reproductive success, and reducing the risk of genetic abnormalities in offspring. The regulation of WIP1 is crucial for the DDR and repair in mammalian oocytes. Recent studies have revealed that the decline in DNA repair capacity in aged oocytes is caused by an increase in WIP1 levels during aging, and WIP1 inhibition could reverse the decrease in DNA repair capacity in aged oocytes, which would improve oocyte quality and developmental competence^139^. Moreover, WIP1 inhibition could reduce sperm-derived DNA damage in fertilized oocytes by enhancing DNA repair capacity^140^. Given the diminished DNA repair capacity of mature sperm, it is plausible that sperm DNA damage is repaired in zygotes via maternal DNA repair machinery^141,142^. Considering the increasing occurrence of DNA damage with aging and the growing use of ART procedures, this research has interesting implications for clinical settings. The therapeutic use of WIP1 inhibitors in ART could be considered, as they could offer a new strategy not only for maternal DNA repair during early mammalian development to ensure the genomic integrity of embryos but also for improving maternal DNA repair capacity in zygotes. Despite several ongoing clinical trials, the clinical efficacy of WIP1 inhibitors has yet to be demonstrated. Therefore, careful consideration should be given to ensure that the therapeutic benefits of WIP1 inhibitors outweigh the risks for patients with infertility, as prolonged periods of WIP1 inhibition might lead to the failure of maturation during early embryo development.

## Concluding remarks and future perspectives

In conclusion, the DDR and DNA repair mechanisms in oocytes are complex areas of study. While the specific mechanisms of DNA damage detection and repair in oocytes are just beginning to be identified, additional extensive studies are needed to fully understand how the oocyte response and repair of DNA damage occur. Most related research, particularly concerning molecular pathways, has been conducted in mouse models, and the insights presented in this review also primarily stem from mouse studies. Therefore, our understanding of oocyte DNA repair in humans is limited. Notably, recent studies have revealed differences in the response to DNA damage between mouse and human oocytes. While DNA damage activates the SAC in mouse oocytes, the same principle does not apply in human oocytes^52^. The reason for the distinct outcomes in mice and humans is unclear. However, gaining a more detailed understanding of the pathway in mice may facilitate a better understanding of the apparent species-specific differences in the DDR of oocytes. Moreover, rapid advancements in high-throughput technology, gene editing techniques, and experimental methodologies hold great promise for enhancing the understanding of DNA damage and repair mechanisms in mammalian oocytes. As these technologies continue to evolve, we anticipate significant progress in unraveling the mysteries of oocyte biology. This ongoing research will not only enhance our theoretical understanding but also pave the way for the development of novel fertility preservation strategies. The implications of such progress are substantial, as they will contribute to safeguarding fertility and addressing the reproductive challenges faced by many individuals. In essence, further investigations in this field will undoubtedly play a vital role in advancing the frontiers of reproductive medicine and preserving fertility. By revealing the secrets of the oocyte DDR and DNA repair, we can offer new hope to individuals seeking to preserve their fertility and overcome obstacles to their path to parenthood. Ultimately, this research carries significance for both individuals and society, as it can be used to shape the future of reproductive health and family planning.
